# Enhancing the collectivist critique: accounts of the human enhancement debate

**DOI:** 10.1007/s11019-021-10030-7

**Published:** 2021-06-16

**Authors:** Tess Johnson

**Affiliations:** Oxford Uehiro Centre for Practical Ethics, University of Oxford, Oxford, UK

**Keywords:** Human enhancement, Genome editing, Individualism, Collectivism, Medical ethics

## Abstract

Individualist ethical analyses in the enhancement debate have often prioritised or only considered the interests and concerns of parents and the future child. The collectivist critique of the human enhancement debate argues that rather than pure individualism, a focus on collectivist, or group-level ethical considerations is needed for balanced ethical analysis of specific enhancement interventions. Here, I defend this argument for the insufficiency of pure individualism. However, existing collectivist analyses tend to take a negative approach that hinders them from adequately contributing to balanced ethical analysis, and often leads to a prohibitive stance. I argue this is due to two common problems with collectivist analyses: inappropriate acceptance of individualist assumptions, and failure to appropriately weigh individual vs collective ethical considerations. To further develop the collectivist critique in the enhancement debate, I suggest we may look to collectivism in public health ethics, which avoids these problems.

## Introduction

Germline genome editing for enhancement (GGEE) consists of causing heritable modifications to the embryo or gamete cell genome (using techniques such as CRISPR-Cas9 editing), for enhancement purposes. To capture public understanding, I conceive of ‘enhancement’ as aiming, not to eliminate genetic predispositions for disease, but to increase or improve an individual’s non-deficient (or normal) functioning for a particular trait, beyond a species-typical level, for their own or others’ benefit.^1^

To date, an individualist approach to assessing the ethical permissibility of GGEE has predominated. I define an ‘individualist’ approach to GGEE as:

Ethical analysis of a GGEE intervention that solely considers the interests and concerns of individuals undertaking the act or directly affected by it, or prioritises them over interests and concerns of third parties.

In this case, the individuals undertaking the act are the parents and their future child. Relevant individualist ethical considerations may include: parents’ reproductive liberty, parental responsibilities, risks and benefits to the future child, and their autonomy.

Here, I first argue that it is problematic to treat the individualist approach as a comprehensive ethical analysis—that is, as decisive of permissibility, rather than as contributing *pro tanto* reasons for or against the permissibility of a given GGEE intervention. This argument aligns with the ‘collectivist critique’, which aims to use collectivist concepts to supplement individualism in ethical analysis.^2^ I define a ‘collectivist’ approach to mirror individualism, as:

Ethical analysis of a GGEE intervention that solely considers the interests and concerns of collectives affected by the intervention, or prioritises them over interests and concerns of the individuals directly involved.

Relevant collectivist considerations may include: the effects of widespread enhancement on community solidarity, public goods-type benefits to the collective, and distributive and social justice concerns. The collectivist critique holds that using these collectivist concepts is necessary for a thorough ethical analysis that weighs both individual-level and collective-level morally relevant considerations regarding GGEE interventions. The collectivist critique may seem misplaced if it claims that individualist work fails entirely to address any collective-level moral considerations. I would agree, and wish to acknowledge that much individualist work is merely *primarily* individualist in the considerations it includes. However, this still leaves room for a legitimate collectivist critique in the sense I intend, so long as there is not an *appropriately balanced* consideration of collective- and individual-level morally relevant concerns. I also argue, however, that current collectivist work requires further development to effectively make its contribution to thorough, balanced ethical analysis. I examine how some current collectivist work (Andorno et al. 2020; Kass 2003; Sandel 2007; Sparrow 2016) demonstrates problems that can lead prematurely to the conclusion that GGEE should be prohibited. Finally, I argue that to further develop the collectivist critique, we may apply concepts from other areas such as public health ethics (PHE), where collectivism has been effectively developed without the problems I discuss.

In Sect. 1, I first introduce individualism in the enhancement debate, identifying areas in which individualism alone cannot adequately incorporate all relevant ethical concerns. I then propose an ideal form of collectivist contribution in Sect. 2, and identify two problems with current collectivist work: failures to challenge individualist assumptions (§2.1), and failures to appropriately weigh individualist *vs* collectivist ethical considerations where they conflict (§2.2). Finally, I argue in Sect. 3 that collectivism in PHE avoids these problems and can thus be appropriately used to model further development toward an ideal collectivist critique in the enhancement context.

Before proceeding, let me defend some initial points.

Collectives (that is, aggregates of the individuals directly involved in GGEE interventions, other specific groups, and the public or society more broadly) may be affected by GGEE interventions in a way that renders collectivist ethical analysis necessary. For example, any use of public funds to subsidise GGEE interventions means that taxpayers are affected by the costs of GGEE interventions, just as they are by taxpayer-supported healthcare or education. Thus, their legitimate collective interest in interventions must be considered in ethical analysis. Collectives may also be affected by virtue of their interactions and connections with radically enhanced individuals, because these individuals may have significantly altered natures and relationships (Fukuyama 2002). This is especially clear when we consider the heritability of germline modifications affecting relationships in future generations, both individually and aggregately across multiple relationships. Finally, some enhancements that are in an individual’s interest, and indeed in multiple individuals’ interests, are not in the group’s collective interest, because of the small negative effects that securing the individual interest has on the group. Such instances may constitute ‘collective action problems’, wherein the seemingly morally insignificant, small detrimental effects on others of a single intervention are much larger so as to be morally significant on the collective scale, when an intervention becomes widespread and the effects are multiplied. The likely popularity of GGEE interventions (given their possible benefits for one’s child) is one reason to take such collective action problems seriously (Anomaly 2018).^3^

These collective-level effects of GGEE interventions cannot in all cases be divided, and reduced to the individual level. Aggregative effects may be possible to divide and distribute among individuals within a collective. But collectives can also be conceived holistically (wherein the collective is treated as having properties emergent only at the group level, and thus as more than the sum of its parts, and affected as a whole entity).

To illustrate, consider ‘public goods’^4^ outside the enhancement sphere that benefit a society as a collective. They may provide only tiny benefit for each individual member of the public (say, a public transport network merely reducing traffic on the road for some drivers), and so, if the benefits are divided and distributed, they seem unimportant for a state to provide. However, they may significantly benefit the society as a whole (say, by the network allowing for city expansion, increased productivity due to less time spent on the road, and lowered carbon emissions that benefit the whole group). In considering only the (less significant) benefits to individuals within a group rather than their sum for the whole, strict individualism does not adequately value public goods. Where this results in states not creating or maintaining those public goods, an individualist analysis may lead to a significant opportunity cost or even significant harm for a society. This holds in the enhancement context. Consider, for instance, cognitive enhancements that increase the public goods of knowledge or productivity in a society. They, similarly, cannot be adequately valued when the benefits of cognitive enhancement to individuals are considered one by one, in comparison to potential costs.

Alternatively, take an example of a collective action problem produced by an enhancement—height enhancement. Parents constantly seeking to have taller children in order to secure the advantages of being comparatively tall for their child will place very small costs on others—say, by adding stress to a healthcare system. This is plausible, if height enhancement were pursued on a population scale, due to the association of tallness with osteo-arthritis and other bone conditions. Ethical analysis on the collective level makes these concerns visible, highlighting not only individual harms to health, but possible collective harms from an over-burdened healthcare system. In providing a further reason against the pursuit of this type of enhancement, a collectivist analysis, as opposed to only an individualist one, better ensures that harmful uses of GGEE will not be permitted.

Collective action problems and the under-valuation of public goods are among the existing problems that individualist ethical analyses face. They serve to highlight the urgency of an effectively-developed collectivist approach, both in ethical analysis of public issues more broadly, and particularly in the case of enhancements.

## Individualism in the human enhancement debate

It would be simplistic to present individualism and collectivism as binary choices of approach. But it seems more plausible that there is a spectrum of approaches between two extremes of pure individualism and pure collectivism. Purer forms of individualism are sometimes traced back to Thomas Hobbes, and his injunction to “consider men as if but even now sprung out of the earth, and suddainly (like Mushromes) come to full maturity without all kind of engagement to each other.” (Hobbes 1651, S8.1). Hobbes and following individualists have considered the self as prior to experience, unencumbered by connection with others.

Contrastingly, more collectivist approaches recognise the self as socially constructed and interdependent with others. Dating back to David Hume, the individualist view of the self as pre-social has met its challenge. Hume claims, contra Hobbes, that “Tis utterly impossible for men to remain any considerable time in that savage condition, which precedes society; but that his very first state and situation may justly be esteem'd social” (Hume 2013 ed., Book III, S2). If the self is socially-constructed, then it follows that group-level ethical considerations cannot be accounted for by reducing the group to its individual components considered separately. Where individual interests overlap, they ought to be aggregated, rather than comparing each of the same interests against a conflicting individual interest separately, in order to recognise their full weight. And where relationships connect individuals within a group, a holistic analysis of ethical considerations relating to the group is required, on this reading.

An example will serve to highlight the difference between more individualist and more collectivist approaches to identifying interests: consider an immune system enhancement that confers immunity against a specific infectious disease (Anomaly 2020; Gyngell and Douglas 2015).^5^ If the disease is prevalent in the community, then it initially appears to be in each individual’s interest to be immune. Therefore, it seems to be in many individuals’ interests that many individuals pursue this enhancement, and therefore in the public interest. However, the public interest depends not only on how immune enhancement affects individuals, but on effects for the group. According to a collectivist approach that acknowledges this, there is a public interest, not in individual immune enhancements as such, but in *a certain number* of individuals being immune in order to maintain herd immunity, *and* a certain number remaining susceptible to the disease. This is because the public also has an interest in maintaining immunodiversity to protect the community from newly emerging diseases to which some people may have genetic immunity.

Take a real-world example. Heterozygotes for sickle cell anaemia are unaffected by malaria due to the sickle cell mutation (Ferreira et al. 2011). This gives sickle cell anaemia (although lethal for homozygotes) adaptive value in areas of sub-Saharan Africa where malaria is prevalent (Piel et al. 2010). For an individualist approach, immunity to a prevalent infectious disease via GGEE intervention is in a child’s interests. Each individual set of parents in a population has a self-interested reason to undertake this intervention for their child (where protecting their child’s health constitutes self-interest). But a collectivist view of the interests involved shows that, were this intervention undertaken universally, significant harms could result for the population, and there is a public interest in the intervention not being too widely pursued. This public interest is not decisive alone, but an individualist approach that excludes it is unable to assess all the interests relevant to permitting pursuit of the GGEE intervention.

The examples below from individualist approaches highlight further the inadequacies of purer forms of individualism. They concern questions of whether individuals should be free to use GGEE as they see fit, whether there should be free market access to GGEE, and what types of interventions ought to be made available.

First, Robert Nozick’s *Anarchy, State and Utopia* (1974) supports a ‘genetic supermarket’ system of GGEE availability. In this system, parents are free to access GGEE if they can afford it, and use it as they see fit. State interference is only justified in order to maintain sex ratios and other essential balances in the human gene pool. The position implies that the development and availability of interventions is acceptably determined by market demand. Individual parents’ abilities to afford and otherwise access in vitro fertilisation (IVF, as procedurally required for GGEE) therefore appropriately determines the distribution of interventions and their benefits. Parents wishing to have children with particular traits are only limited in this choice by their abilities to access IVF. Problems with this approach arise if we recognise the significant effects that GGEE may have on people’s abilities to pursue their life goals and interact with others as moral equals. For instance, in a market system, some Rawlsian ‘primary goods’ (including basic rights, income, and social recognition, which aid in the pursuit of various conceptions of the good life) may be distributed across society according to existing trends of dis/advantage. However, because of the importance of these goods, Rawls argues they ought to be distributed equally, except where unequal distribution benefits the worst-off (Rawls 1999 ed.). If we accept this, then perhaps primary goods-producing enhancements should be distributed by the state. An approach that prioritises individual freedoms, however, may not allow for such intervention. Alternative, more collectivist approaches than Nozick’s might consider the fair distribution of primary goods-producing enhancements to justify some impositions on individual freedoms in order to reduce (or at least not exacerbate) unfair inequalities.

Similarly, with regard to the availability of enhancements, Nicholas Agar’s *Liberal Eugenics* (2004) and, to some extent, Nick Bostrom’s *Human Genetic Enhancements* (2003) each assume that it is ethically acceptable for most enhancements to be sold to individuals, in the same way that we usually accept by default free market access to new technologies. Although Agar does not claim *all* enhancements should be on the free market, his exclusion of only ‘competitive enhancements’—that is, enhancements that benefit the individual only relative to others, rather than intrinsically—from the market system still does not adequately address concerns at the collective level. To illustrate, take his example of a memory enhancement in the job market context. Agar recognises the plight of unenhanced individuals who are disadvantaged in their competition against an enhanced individual for a job. He argues that parents should be “prevented from seeking enhancements that prepare their children exclusively or principally for winner-takes-all competitions” (Agar 2004, p. 130). As he suggests, one option is prohibition. For an alternative simple fix to this problem, one might suggest these enhancements should be subsidised or provided for the worst-off. But provision in fact does not solve the problem. A more collectivist approach recognises that entire groups may be pressured out of the job market by widespread enhancement, not only because of inabilities to access enhancements, but even with universal provision, because the actual benefit of an enhancement may vary for different groups, tracking existing unfair social dis/advantage. Even if memory enhancements were universally provided, they may provide less benefit in the manual and menial jobs that are often filled by already-disadvantaged members of our society, compared to other, more desirable work that is often undertaken by the already-advantaged. For a textile manufacturing job applicant, access to memory enhancement is likely to be less valuable than for those (likely advantaged) individuals applying for, say, a high-level job on Wall Street. If a stocks analyst will be benefited by the availability of memory enhancement, whereas a factory labourer will not, then when this trend is multiplied as such interventions become widespread, the group of advantaged individuals who are more able to compete for Wall Street jobs is further aided in securing the advantages that such work provides, whilst those already disadvantaged groups often applying for manufacturing jobs, are not helped by memory enhancements in being more successful in their work and reaping the benefits this produces. Thus, even universal provision of the memory enhancement may reinforce patterns of existing disadvantage in society when competitive enhancements are made available to individuals. Although, in this case, Agar advocates for simple prohibition of competitive enhancements, his and others’ work still highlights an inadequacy of individualist approaches to GGEE distribution. Namely, they do not adequately recognise variable benefits of GGEE distribution among existing groups—or potential collective-considering solutions. Rather than prohibition, or even universal provision, a thoughtful collectivist analysis might recognise and explore the variable value of memory enhancements for different groups. It could then propose regulatory alternatives that, say, prioritise the provision of primary goods-type enhancements that will benefit society more broadly, before making other selectively- or relatively-beneficial enhancements available.

## Collectivist arguments in the human enhancement debate

Earlier, I said that the collectivist critique aims to contribute to a thorough, balanced ethical analysis of GGEE interventions. In this section I contrast an ideal critique with current collectivist contributions. I argue that the latter are biased toward weighing costs, losses and risks of enhancement more heavily, affecting their ability to contribute meaningfully to the collectivist part of balanced ethical analyses. Underlying this negatively biased collectivism are certain assumptions and processes. These are examined below, in terms of the inappropriate acceptance of individualist assumptions (§2.1), and the failure to appropriately weigh individualist *vs* collectivist ethical considerations where they conflict (§2.2). To solve these problems, we may employ collective-considering moral principles and concepts from other areas such as PHE, which include positive ethical considerations (such as whether an intervention may provide collective benefit, ameliorate existing injustices, or produce public goods).

First, let me provide the standard for contrast: an ideal collectivist critique. Consider a memory enhancement—specifically, a GGEE intervention that improves memory capacity and retrieval beyond species-typical functioning in an individual who would otherwise have normal memory functioning. Individualism might contribute to ethical analysis here by assessing concerns surrounding the authenticity of the child’s altered nature, the side-effects of an intervention that may prevent forgetting, or the tension between, on the one hand, parents’ rights to independent reproductive choice, and on the other, the child’s future wellbeing and right to an open future. An ideal collectivist critique would build on this, perhaps by assessing how widespread enhanced memory could affect important public goods—by promoting knowledge and technological advancement, but threatening the public’s social cohesion, due to a reduced ability to forget others’ past wrongdoings. Reduced social cohesion, involving relationships toward others in a group, certainly cannot be adequately valued by considering effects merely on individuals. It may also assess how these effects ought to be distributed, not only among individuals, but across current and future groups. To illustrate some collective-level considerations, imagine a scenario in which a memory-enhanced generation adds value in workplaces at a much younger age. The enhanced group may end up being exploited, possibly counteracting the individual benefits they experience. On the other hand, consider how enhanced memory may instead actually promote social cohesion, and strengthen relationships in future generations, if having clearer memories of others’ lives and suffering increased empathetic response.

Some current collectivist arguments addressing memory enhancement and other interventions neglect benefits and interests in enhancing (as opposed to its risks and costs), like those considered in the ideal analysis above. Whilst these analyses remain collectivist in nature, they are less effective at contributing, because they are unbalanced. This is partly the product of the following problems, which I explore in turn below.

### Acceptance of individualist assumptions

Some current collectivist analyses accept—either as undesirable but inevitable circumstances, or as morally acceptable—assumptions that decisions surrounding GGEE will be made individually by parents, without state interference, and that distribution and development of GGEE interventions will be governed by the free market. It may seem counterintuitive for current collectivist work to accept these circumstances—public education and public health are areas of policy that are successfully and ethically appropriately regulated by the state. Yet the acceptance persists, and combines with a primarily negative view of collective-level considerations to frequently produce prohibitive stances in the literature.

Michael Sandel’s work, for instance, assumes parents will inevitably (though inappropriately) be left to choose among GGEE interventions without adequate regulatory limits to their decisions, if enhancement is permitted. In *The Case Against Perfection* (2007), Sandel’s assumption that enhancement would be set in a free market paradigm partly justifies his emphasis on potential negative outcomes of GGEE for the collective. He considers challenging the market availability assumption, but claims that remedying unfairness in access to enhancements is not the main issue, though it could be remedied through subsidisation. Sandel’s main focus, rather, is on the loss of openness to the unbidden if enhancements become widespread, and on issues with ‘playing God’. He also discusses the apparent problem of social pressure to enhance, claiming that rather than focussing on changing the market paradigm, “[t]he real question is whether we want to live in a society where parents feel compelled to spend a fortune to make perfectly healthy kids a few inches taller.” (Sandel 2007, p. 18–19). This leads him to argue for prohibition of enhancement. If the emphasis here is on social norms rather than the ‘fortune’ that parents may have to spend in a market system, then subsidisation does initially seem like a futile solution. However, we might question the premise: Sandel’s argument concerning social norms is not independent; it is predicated on our accepting his other arguments against enhancement. These explain why social norms are harmful. But if we acknowledge the potential benefits of enhancement, social norms regarding enhancement may not themselves be a bad thing. Sandel considers social norms discussed in the quote above only from a negative-collectivist perspective, as a necessarily detrimental outcome of enhancement, based on his previous arguments against it. But social norms themselves can be positive (Anomaly and Brennan 2014). If enhancement is not problematic for other reasons, then parents being socially pressured to pursue it at minimal cost, in a subsidised system, may be a positive thing, encouraging them to intervene to benefit their future child and/or the collective.

Providing a second example, Leon Kass’ arguments against GGEE (2003) also frequently assume a free-market paradigm. His claim is that in American society at least, if enhancement is permitted, the emergence of an enhanced aristocracy seems inevitable, as “there is nothing in our current way of doing business that works against it” (Kass 2003, p. 282). Kass does not imply that such a situation is desirable, but he does seem to see it as unavoidable. This fails to consider existing areas of effective state interference, most notably in public health, defense and education that ensure the important goods of health, safety and educational opportunity are fairly accessible. In adhering to the free market assumption, Kass does not consider regulatory alternatives (perhaps including subsidisation, prioritised access, and limits on the development of certain interventions) that may even allow enhancements to be used to *address* existing injustices, rather than exacerbating them (Giubilini and Minerva 2019). Based on the individualist assumption he accepts, negative collective-level ethical considerations seem more salient, and lead Kass to argue for prohibiting enhancement. This approach to potential intergenerational fairness concerns with enhancements is mirrored by some of Robert Sparrow’s work (2019). Sparrow contends that “If the genetic enhancements available to parents to choose for their children improve every year, then the enhancements provided to children in any given year will quickly become obsolete” (Sparrow 2019, p. 8). Whilst raising important issues concerning intergenerational justice, this concern is based on the assumption that parents will be left to choose between available enhancements freely, and that the value of enhancements is not absolute, but relative to those that may come later. That is, that children are benefited in terms of their advantage in competitive environments, rather than being benefited by the absolute value of any enhancements that aim to produce group-level benefits outside of competitive contexts.

Similarly, Roberto Andorno and colleagues’ recent *Geneva Statement on Heritable Human Genome Editing* (2020) also initially assumes a free market. The authors examine a selection of possible outcomes of genome editing including state eugenic uses of GGEE, widespread genetic harm to future generations, competitive pressures, and exacerbations of racism and xenophobia. Their discussion of these problems is predicated on the assumption that adequate regulation is implausible, yet they do not examine regulatory alternatives or assess corresponding possible positive outcomes, which ought to figure into a balanced analysis. Non-coercive policies encouraging uptake of collectively beneficial interventions (such as free exercise programmes or letters encouraging cancer screening) are evident already in the public health context, and might apply for enhancements as well. For example, states might subsidise interventions that enhance IQ-measured intelligence in order to increase productivity and knowledge in a society. The authors’ arguments, emphasising negative aspects of analysis over positive, offer an incomplete analysis. Although positive considerations may not outweigh the negative overall, we must still give them consideration, and not base discussion only on assumptions that make the negative more salient.

### Failure to appropriately weigh collective- and individual-level considerations

Some current collectivist work suffers from the second problem that may lead to negatively biased collectivism: failure to adequately (or equally) weigh collectivist considerations in overall analysis, compared to individualist considerations. This may occur even where harms or costs to individuals of GGEE interventions that are collectively beneficial are seemingly easily avoidable.

Some of Robert Sparrow’s work (2016) assumes that collectivist considerations will, if GGEE is permitted, consistently override individual interests and result in unacceptable eugenic practices. He explicitly concludes that some collective interests in genome editing or selection should not figure in ethical analysis at all, for fear that “considerations of aggregate welfare in decisions about reproduction […] threaten to outweigh any of the other interests at stake” (Sparrow 2016, p. 134), and would result in unacceptable infringement of individual liberty. This claim assumes that collective interests, if they are to be considered at all, must be determinative of the permissibility of GGEE, simply in light of their affecting many more people. In that case, potentially very significant costs to individuals may apparently be imposed for the sake of a collective interest in genome editing. Sparrow proposes, therefore, that these collective interests are best rejected altogether. Otherwise, “[g]iven the number of third parties who might benefit from the selection of future individuals that have particular sorts of capacities, there is little that the concern for the interests of such third parties could not justify” (Sparrow 2016, p. 134). He argues that such interests would be governed, in the end, by nationalistic state goals.

However, this slippery slope argument is not convincing if there are definite, appropriate limits incorporated into a system for weighing ethical considerations that ensures collective interests are weighed against individual interests (where they conflict) in a proportionate way. In some cases, this will mean that collective interests are not significant enough to outweigh individual interests. For example, imagine a ‘social compliance’ enhancement that increases contentment in populations and offers greater societal stability. It may also significantly threaten the enhanced individuals’ authenticity and integrity. This may be one case in which the interest in authenticity and integrity, even considered on an individual basis, outweighs the collective interest, given how integral these values are to our identities. In other cases where more significant collective interests pertain and less significant individual concerns conflict with them, we may be justified in imposing some small costs on individuals in providing state support for GGEE interventions. For example, a moral enhancement that increased our rational capacities or our empathy and produced fairer, more solidaristic societies generally may be acceptable. It does, by hypothesis, pose a small cost to individuals in terms of contributions for state funding of the intervention. Whilst this may seem unacceptable to individual-prioritising accounts of enhancement, according to a collectivist account this imposition of costs may be acceptable, if the collective-level benefit is much greater than the cost to individuals.

To address the weighing concern more generally, collective-level interests will only outweigh individual-level concerns in a proportionate weighing system when the individual costs are either:

- morally insignificant (such as in cases of easy rescue),
- plausibly avoidable by opting for a less coercive way of implementing the intervention, or
- acceptable in the proportion of costs they impose on individuals *vs* benefits they produce for the collective.

This weighing system that I propose applies in the same way to individual-level interests and collective-level costs. In some cases, weighing costs and interests exposes the disproportionate risk that prioritising the collective may pose to individual wellbeing or liberties. Consider, for example, threats to autonomy (for women who might otherwise be fined for non-compliance with a mandatory enhancement programme) or bodily integrity (if such women were instead physically forced to have IVF for enhancement). However, where such morally significant harms are either proportionate to the benefit produced, or avoidable by avoiding compulsory (or otherwise coercive) implementation of the intervention, the weighing system may show the intervention to be permissible.

Take compulsory immunity enhancement via GGEE. This would pose a significant threat to the bodily integrity and autonomy of women forced to undergo invasive IVF treatment as part of the intervention. However, this does not imply that the public interest in herd immunity should be disregarded *tout court* because of the bodily integrity and autonomy threats: it must be still considered, and it must be determined whether the collective interest poses an adequate benefit to justify the individual cost, rendering the compulsory intervention permissible. In this case, I suspect it would not, as many women would likely have their bodily integrity significantly compromised for the sake of this public good that may alternatively be promoted via vaccination. It may be that a way of implementing the intervention that avoided the threat to individuals by *incentivising* it rather than making it compulsory is acceptable. This weighing system would not allow collective considerations alone to determine permissibility.

There is another problem in the collectivist literature with weighing ethical considerations: weighing concerns differently according to whether genome editing is performed for treatment or enhancement purposes.^6^ Francis Fukuyama (2002) weighs (individual) benefits more heavily when it comes to treatment interventions, but discusses and weighs (collective) risks and costs more heavily when it comes to enhancement interventions. He does not offer a reason why threats to the collective are more significant for enhancement than treatment. His view of an enhanced future leads him to reject the possibility of enhancement regulation as an implausible solution to collective-level problems with enhancement, but it is little considered how editing for *treatment* purposes might negatively affect the collective, or how editing for enhancement purposes might positively affect the collective. For enhancement, Fukuyama claims we risk losing ‘Factor X’, a defining characteristic of humanity that underlies our commitment to human rights and dignity. This will “in some way cause us to lose our humanity—that is, some essential quality that has always underpinned our sense of who we are and where we are going.” (Fukuyama 2002, p. 101). In the treatment case, however, the threat to Factor X is apparently insignificant. However, there is no reason given for why the threat to the collective would be more significant in the case of enhancement (or the benefit to the individual more significant in the case of treatment). Surely, whether enhancement threatens to undermine valuable aspects of humanity depends on the types of enhancement being considered. An alternative, collectivist consideration of human dignity goes further than Fukuyama’s Factor X in detailing specifically how we can justify some collectivist concepts that may constrain our pursuit of enhancements. As “one of the fundamental values of our community”, ‘human dignity as constraint’ has been developed to limit the appropriate pursuit of biotechnologies (Beyleveld and Brownsword 1993, p. 30). These limits are based not primarily on individual rights, but in consideration of duties to protect others’ and our shared dignity. Applying this concept demonstrates how enhancements may *not* threaten our humanity or human dignity more than treatments. This is clearer when considering dignity, compared to Fukuyama’s concept. It is difficult to judge, for instance, whether increasing memory capacity threatens Factor X more than the treatment of congenital deafness that could result in the elimination of sign languages and Deaf culture. But when we consider human dignity as constraint, it seems clearer that memory enhancement does not pose a significant threat to human dignity, whereas the elimination of a valuable culture via GGE for treatment may do so. We might think that Deaf culture is more valuably human than a limited memory capacity. Correspondingly, it is unclear whether the benefit of being born hearing is necessarily greater in some contexts than the benefit of being born with much better memory. This inconsistency in weighing costs and benefits across enhancement and treatment interventions leaves Fukuyama’s analysis wanting.

## Public health ethics collectivism and relevance to the enhancement debate

Further development of the collectivist critique is required if we are to avoid the two problems discussed above, and the negative analysis that they lead to. Collectivist work in public health ethics (PHE) provides examples of success, where it contributes effectively to individual-collective balanced ethical analysis for policymaking and employs moral principles and concepts in analysis that can be used to the above problems identified with collectivism in the enhancement sphere. Whilst collectivism in PHE is itself problematic in some ways, where it has concepts that fill the gaps in enhancement collectivism, it provides a useful area from which lessons can be drawn. It is outside the scope of this work to critique PHE collectivism. Nevertheless, it seems plausible to say that there is an analogy between enhancement and public health interventions, when these aim to benefit a population’s wellbeing. I propose, then, that collectivist moral concepts and principles from the one area may be appropriately applied in the other. Healthcare systems themselves constitute public goods that provide collective benefit, whilst imposing costs via taxation in order to maintain them. Similarly, I have discussed how some enhancement interventions may impose some acceptable individual or collective costs whilst providing similar wellbeing-type benefit.

In order to articulate the collective-level considerations at stake, relevant concepts used in PHE collectivism can be applied to the enhancement debate. It is mostly with regard to public health problems involving public interests or widespread health outcomes, and where collective action may be required, that we see collectivist concepts being usefully applied in PHE.^7^ These areas include, for example, obesity and vaccination interventions, both of which I use below to demonstrate how relevant collectivist concepts enhance collectivist elements of ethical analysis in PHE, and may similarly do so in the enhancement context.

Research that has focused on structural (that is, social, economic, and environmental) causes of obesity in children has shown the ineffectiveness of individual-centred approaches in combatting obesity whilst corporate interests in marketing sugary foods to children continue to prevail (Lobstein and Dibb 2005). Ethical analyses building on similar studies in PHE have developed robust conceptions of collective responsibilities for health outcomes (Brown et al. 2019). They suggest we may have a collective responsibility to ensure our environments are structured to promote good health choices, rather than individual choices that lead to obesity (in this case). In environments that make healthy choices very difficult, individuals cannot be held fully responsible for choices that produce negative health outcomes (Brown et al. 2019). This is especially the case where children’s responsibilities are concerned. Fran Baum (2019) discusses how issues such as obesity are more effectively and more ethically appropriately targeted using a collective-level measures that either better hold corporations and other groups accountable, or limit their power in health contexts, as part of recognising a collective level of responsibility for negative effects on individuals’ health choices. She also notes the accumulating evidence for a correlation between levels of social inequality and health outcomes, wherein “societies in which there are more income inequalities between people also have […] higher rates of obesity, […] and reduced educational attainment and social mobility” (Baum 2019, p. 29).

Where we recognise a robust collective-level responsibility for certain health decisions, we can better address factors that compromise health outcomes. In the example case, this means increased corporate accountability for the health effects of advertising unhealthy foods to children. In the case of enhancement interventions, the application of a robust concept of collective responsibility may allow for more thorough assessments of how widespread uses of an enhancement affect social norms, influencing individual decision-making surrounding GGEE interventions. Additionally, it may help in the identification of existing collective responsibilities that create collective interests in certain GGEE interventions which could help fulfil those responsibilities. This allows us to recognise collective benefits more clearly. For example, moral enhancements that made members of society more altruistic may aid in fulfilling a collective responsibility to reduce existing unfair inequalities in a society (Persson and Savulescu 2012).

In the case of vaccination, PHE analysis again puts collectivist concepts to good use in the development of a balanced approach. This area employs the concept of public goods particularly effectively, concerning the maintenance of herd immunity. Herd immunity is a public good that protects anyone and everyone in a community from an infectious disease. The benefits of vaccinating one’s child are thus not only individual, but serve the collective as a whole. However, interests can conflict where vaccination poses risk to the individual. The collective interest in maintaining herd immunity, thus preventing excess mortality or stress on healthcare systems, must be weighed against the individual costs of the discomfort of being vaccinated (Giubilini et al. 2018). Here I assume that where an adverse reaction to a vaccine can be expected, such individuals are not asked to comply with vaccination. For those who will experience mere discomfort, given the importance of herd immunity, the concept of duties of ‘collective easy rescue’ may be applied, wherein contributions to public goods or collective benefits that impose only small individual costs are appropriately enforced (Giubilini and Savulescu 2019). Policies in some jurisdictions reflect this by disincentivising non-vaccination, imposing costs on parents who do not have their children vaccinated, such as limited access to child support financial benefits (Commonwealth Government of Australia 2016). Where non-contribution to public goods such as herd immunity threatens the collective interest and allows individuals to avoid only an insignificant cost, an adequately collective-considering and appropriately weighed ethical analysis justifies limited coercive government interference. This same concept, if applied in the enhancement sphere, may allow for further articulation of the proportionate weighing of collective and individual interests, permitting cases in which individual interests and collective interests are of a comparative significance such that the collective interest greatly outweighs an insignificant individual cost. GGEE interventions may maintain or promote public goods such as knowledge, safety, health, and political participation. Where they do so at an insignificant cost to individuals, they may be permissible (or even, in some cases where other considerations don’t pertain, enforceable). Cognitive enhancements may increase the production of knowledge; moral enhancements may provide means to promote safety in society—the collective interest in these significant goods must be appropriately compared to the individual costs they involve. There is a public interest in these goods, which ought not be ignored in a balanced analysis—and need not be, as the successful use of concepts in the PHE sphere shows.

## Conclusion

In order to complement an individualist perspective in balanced ethical analysis of enhancement interventions, collectivist work as it stands needs further development. As demonstrated by some collectivist arguments, two common problems can lead to negatively biased analyses. These problems are: first, failures to challenge individualist assumptions, and second, failures to appropriately weigh collectivist *vs* individualist considerations at stake. However, using collectivist work in PHE as a model, we may apply collectivist ethical concepts to the enhancement debate, and enhance the collectivist critique as it stands. The resulting enhanced critique can better inform a balanced framework for ethical analysis of GGEE interventions.

## Footnotes

1 Each element of this definition is required in order for an intervention to count as an enhancement, thus excluding non-beneficial disease-unrelated modifications like, say, hair colour modifications (in some contexts). For further discussion, see Segers et al. (2019).

2 I acknowledge that collectivism can be understood in various ways. For example, in the case of religious groups, to refer to the interests or commitments of their own community, rather than the interests of a broader society. Thus, we can distinguish between broader collectivism, and what we might call this form of ‘community collectivism’. However, I use the former, broad understanding.

3 For further discussion of the “liberal dilemma” that states face concerning conflicts between individual and collective interests, see Nielsen et al. (2001).

4 That is, resources in a society that are accessible by anyone (non-exclusive) and that are not depleted by a number of individuals trying to access the good at once (non-rivalrous).

5 Insofar as my definition is concerned, this constitutes enhancement by virtue of its: 1) not eliminating a predisposition to a disease, 2) modifying a normal individual’s immune function, and 3) producing benefits. I acknowledge that to other definitions, this constitutes a grey area between enhancement and treatment.

6 Note that this approach aligns with a conceptual argument concerning the treatment-enhancement distinction. Adherents to the distinction hold that there is a descriptive and normative difference between treatment and enhancement. Whilst treatment-uses of genome editing are acceptable, enhancement uses are not, because treatments aim to correct for existing deficits, whereas enhancements do not. I do not engage with this or other, similar conceptual arguments surrounding therapy vs. betterment, discussions of naturalness, or definitions of disease. For further discussion of these, see Hofmann (2017).

7 Here, I examine collective-oriented public health ethics concepts, aligning with a collectivist critique. However, it should be noted that other areas of health justice are successfully grounded on more individualist, but non-libertarian concepts that similarly allow for balanced debate. The capabilities approach is one such method. Due to space limits I cannot explore the full scope of such approaches here.
